# MDAKRLS: Predicting human microbe-disease association based on Kronecker regularized least squares and similarities

**DOI:** 10.1186/s12967-021-02732-6

**Published:** 2021-02-12

**Authors:** Da Xu, Hanxiao Xu, Yusen Zhang, Mingyi Wang, Wei Chen, Rui Gao

**Affiliations:** 1 School of Mathematics and Statistics, Shandong University, Weihai, 264209 China; 2 Department of Central Lab, Weihai Municipal Hospital, Cheeloo College of Medicine, Shandong University, Weihai, Shandong China; 3 School of Control Science and Engineering, Shandong University, Jinan, 250061 China

**Keywords:** Association prediction, Microbe, Disease, Machine learning, Kronecker regularized least squares

## Abstract

**Background:**

Microbes are closely related to human health and diseases. Identification of disease-related microbes is of great significance for revealing the pathological mechanism of human diseases and understanding the interaction mechanisms between microbes and humans, which is also useful for the prevention, diagnosis and treatment of human diseases. Considering the known disease-related microbes are still insufficient, it is necessary to develop effective computational methods and reduce the time and cost of biological experiments.

**Methods:**

In this work, we developed a novel computational method called MDAKRLS to discover potential microbe-disease associations (MDAs) based on the Kronecker regularized least squares. Specifically, we introduced the Hamming interaction profile similarity to measure the similarities of microbes and diseases besides Gaussian interaction profile kernel similarity. In addition, we introduced the Kronecker product to construct two kinds of Kronecker similarities between microbe-disease pairs. Then, we designed the Kronecker regularized least squares with different Kronecker similarities to obtain prediction scores, respectively, and calculated the final prediction scores by integrating the contributions of different similarities.

**Results:**

The AUCs value of global leave-one-out cross-validation and 5-fold cross-validation achieved by MDAKRLS were 0.9327 and 0.9023 ± 0.0015, which were significantly higher than five state-of-the-art methods used for comparison. Comparison results demonstrate that MDAKRLS has faster computing speed under two kinds of frameworks. In addition, case studies of inflammatory bowel disease (IBD) and asthma further showed 19 (IBD), 19 (asthma) of the top 20 prediction disease-related microbes could be verified by previously published biological or medical literature.

**Conclusions:**

All the evaluation results adequately demonstrated that MDAKRLS has an effective and reliable prediction performance. It may be a useful tool to seek disease-related new microbes and help biomedical researchers to carry out follow-up studies.

## Background

With the fast development of advanced analytical techniques and high-throughput methods for exploring complex microbial communities, in human disease and health, the role of the microbiome has gained widespread attention over the past decade [1, 2]. The microbial community is complex and immensely diverse, research showed that about 100 trillion archaeal and bacterial cells in the human gut which belong to more than 1000 species, tenfold the number of human cells [3, 4]. Microbes are closely related to human health and disease. Generally, most of the gut microbes are either harmless or even beneficial to the human body, such as which can contribute to normal immune function, improve metabolic capability and protect against enteric pathogens [5, 6]. Therefore, microbes are also considered as “forgotten organs” in host [7]. But, if the normal balance between the host and microbiota is broken, which may possibly induce many diseases, including asthma [8], inflammatory bowel disease (IBD) [9], brain disorders or neurodevelopmental deficits [10] and even cancer [11], and so on.

Unquestionably, it has a great significance to identify microbes related to diseases for revealing the pathological mechanism of human diseases and understanding the mechanisms of microbe-host interactions. Some large-scale projects have been initiated, such as the Human Microbiome Project (HMP) [12] and Metagenomics of the Human Intestinal Tract of European Union Project (MetaHIT) [4, 13]. It can help us to initially understand the significance of medicine and biology, functional states and healthy composition of the human microbiome [5]. It is still a challenge to understand how the microbiome influences human diseases, since the microbial community is complex and diverse. Effective computational methods could significantly reduce the time and cost of traditional culture-based microbial experiments. Researchers could select potential MDAs for experimental verification. In 2016, Ma et al. [14] manually collected and developed a human microbe–disease association database (HMDAD), which provided the foundation for identifying the MDAs through computational methods.

In general, we could transform this biological problem of predicting disease-related microbes into a link prediction task. In fact, some computational methods have been widely developed to solve the association or interaction problem such as miRNA-disease [15], drug-target [16], lncRNA-protein [17] and protein–protein interaction [18] prediction problems, and so on. However, to the best of our knowledge, until 2016, there are almost no related MDAs prediction researches from a computational point of view. Thereafter, in 2016, Chen et al. [19] designed the first computational method called KATZHMDA for the prediction of MDAs. It is a KATZ measure-based network prediction method to solve the problem of MDAs prediction by calculating the Gaussian interaction profile (GIP) kernel similarity. Beyond that, in recent years, some network-based methods were also proposed only using the GIP kernel similarity for prediction, which are primarily based on the fusion of known associations and heterogeneous data to construct the network, including random walking-based methods [20, 21], label propagation-based method [22], path-based method [23]. In 2017, Huang et al. [24] presented the NGRHMDA method by integrating two single recommendation methods (graph‑based scoring and neighbor‑based collaborative filtering prediction model), and achieved a good prediction result. With the fast development of machine learning technology [25, 26], some machine learning-based methods were also presented for MDAs prediction. For example, in 2017, Wang et al. [27] proposed a semi-supervised method called LRLSHMDA based on the Laplacian regularized least squares method. In addition, in 2018, He et al. [28] and Shi et al. [29] developed machine learning-based method named GRNMFHMDA and BMCMDA for MDAs prediction, respectively, based on the graph regularized non-negative matrix factorization and binary matrix completion.

In recent years, the above computational methods mainly utilized a basic assumption that microbes with similar functions will share similar non-interaction or interaction patterns with phenotype diseases [30, 31]. With the fast development of machine learning technology, the regularized least squares algorithm is a useful tool and has been widely used in the recommended system [32–34]. Although some computational methods have been developed, most disease-related microbes remain unknown and effective methods are still scarce [5, 35]. We could address or reduce some limitations to improve the prediction performance of the computational method. For example, some existing methods only used the GIP kernel similarity for extracting the efficacious information, which may lead to the algorithm inevitably biased against well-researched microbes and diseases, multivariate information fusion will be more helpful for prediction. Beyond that, some existing methods did not consider that the effective contribution of diseases and microbes is uneven due to the number of diseases and microbes is different in the database [36]. It is necessary to improve calculation speed since some methods integrate multiple calculation methods which may be complex and time-consuming. Some methods used many model parameters which may reduce robustness and do not apply to new data.

In this paper, considering some of the above limitations, we developed a novel computational method called MDAKRLS based on the Kronecker regularized least squares method to identify potential MDAs. It is a machine learning-based method and uses fewer model parameters, which can save time and obtain robust performance. First, we calculated Kronecker Gaussian similarity and Kronecker Hamming similarity of microbe-disease pairs based on the known microbe-disease association network. Then, the Kronecker regularized least squares algorithm used two different Kronecker similarities to obtain prediction scores, respectively. Finally, we obtained the final prediction results by integrating the contributions of different similarities. The experimental results of 5-fold cross-validation (5-CV) and global leave-one-out cross-validation (LOOCV) indicated that MDAKRLS can achieve superior performance by comparing it with five state-of-the-art methods. In addition, case studies further demonstrated that MDAKRLS is a useful tool that can effectively identify potential MDAs.

## Materials and methods

**Fig. 1:**
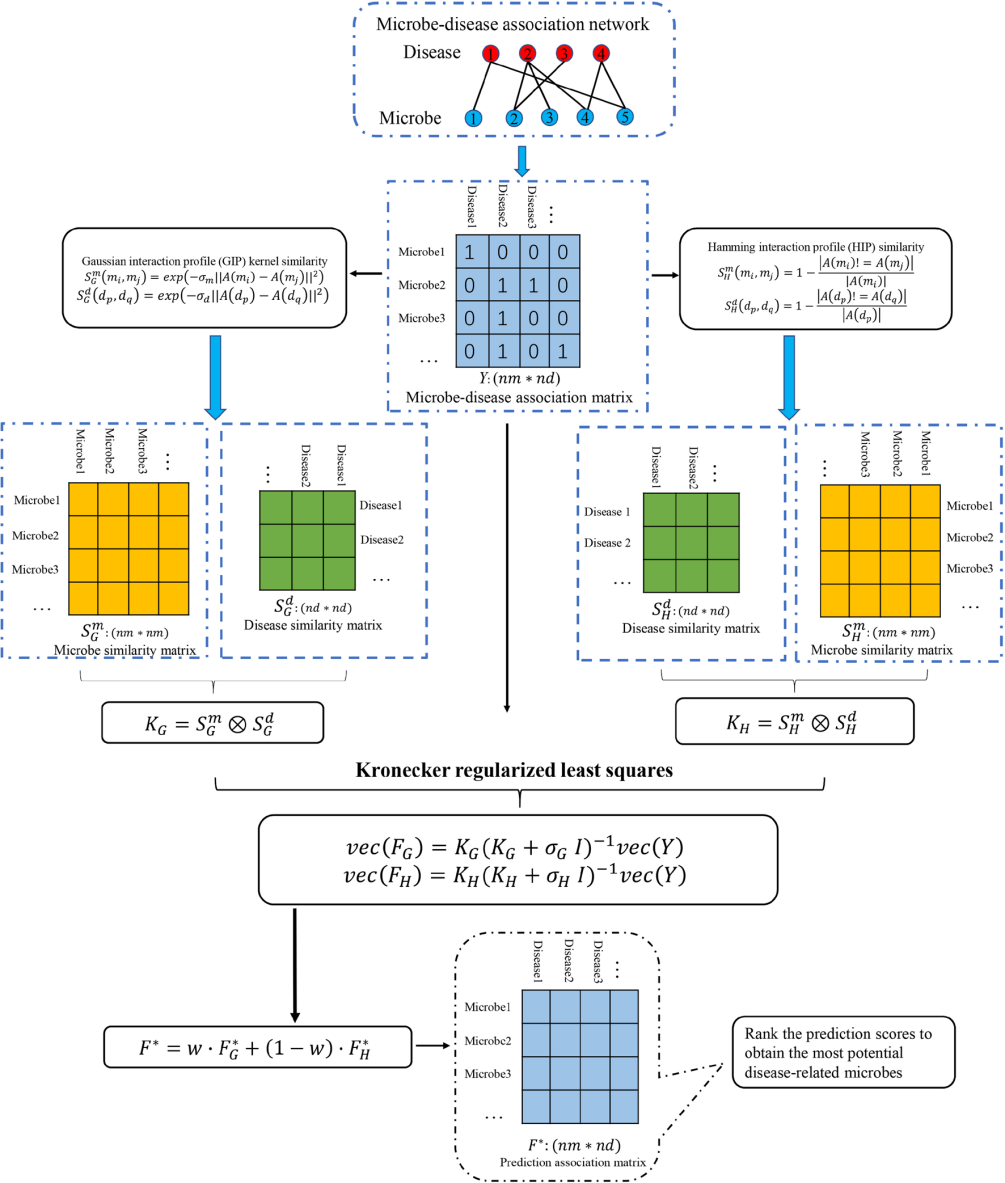
Overall workflow of MDAKRLS applied to human MDAs prediction

In this work, we proposed a novel method called MDAKRLS for inferring latent MDAs. Figure 1 describes the overall flow chart of MDAKRLS for prediction. The framework of prediction method consists of three steps. First, we constructed Kronecker Gaussian similarity ${K}_{G}$ and Kronecker Hamming similarity ${K}_{H}$ of microbe-disease pairs by fully exploiting Gaussian interaction profile (GIP) kernel similarity and Hamming interaction profile (HIP) similarity from known microbe–disease association matrix, respectively. Second, we introduced the Kronecker regularized least squares algorithm based on two Kronecker similarity to construct loss function for prediction. Third, we used an integration strategy to get the final predicted association matrix. Finally, the final possibility score of each microbe-disease pair can be calculated.

### Human microbe–disease association data set

In this study, we used a widely-used benchmark data set (HMDAD) to evaluate the reliability and effectiveness of MDAKRLS. It was manually collected by Ma et al. [14] and can be available at http://www.cuilab.cn/hmdad. The database contains a total of 483 verified associations, 292 human microbes and 39 diseases. The microbe-disease association data set adopted by us was downloaded from HMDAD in June, 2020. We finally obtained 450 verified associations after we removed repetitive associations. In fact, we represented the advantages of MDAKRLS through the overall HMDAD data set. For a better description, we constructed an adjacency matrix $A \epsilon {R}^{39\times 292}$ to express the associations network.

### Similarity measures

For a better description, in this study, set $D=\left\{{d}_{1},{d}_{2},\dots ,{d}_{i},\dots {d}_{nd}\right\}$ and $M=\left\{{m}_{1},{m}_{2},\dots ,{m}_{j},\dots ,{m}_{nm}\right\}$ denote the sets of diseases and microbes, respectively. We introduced an adjacency matrix $A \epsilon {R}^{nd\times nm}$ to express the associations network, where variable $nd$ denotes the numbers of diseases; $nm$ represents the numbers of microbes. Besides, the adjacency matrix $A \epsilon {R}^{nd\times nm}$ is defined as follows:

1 $$A\left(i,j\right)=\left\{\begin{array}{c}1, \quad if \, disease \, {d}_{i} \, is \, related \, to \, microbe \, {m}_{j}\\ 0, \quad otherwise\end{array}\right.$$

Set $A\left({d}_{i}\right)\epsilon {\{0, 1\}}^{1*nm}$ represents the $i$ th row of $A$, which is a binary vector and denotes the interaction profile of the disease ${d}_{i}$. Similarly, $A\left({m}_{j}\right)\epsilon {\{0, 1\}}^{nd*1}$ denotes the $j$ th column of $A$, which represents the interaction profile of the microbe ${m}_{j}$. According to the basic assumption, microbes with similar functions will share similar non-interaction or interaction patterns with phenotype diseases, which is widely used in the related studies. To integrate more effective information and uncover potential associations, we calculated the GIP kernel similarity and HIP similarity of human microbes and diseases, respectively.

#### GIP kernel similarity for microbes and diseases

To mine conveniently the topological structure information of association matrix $A$, we used the GIP kernel similarity [19, 37] for measuring similarity of human microbes. Specifically, for two given microbes ${m}_{i}$ and ${m}_{j}$, we first extracted their interaction profiles $A\left({m}_{i}\right)$ and $A\left({m}_{j}\right)$ from the training adjacency matrix $A$, respectively. Subsequently, the GIP kernel similarity of microbes can be calculated as follows:

2 $${S}_{G}^{m}\left({m}_{i}, {m}_{j}\right)=exp\left(-{\sigma }_{m}{||A\left({m}_{i}\right)-A\left({m}_{j}\right)||}^{2}\right)$$

3 $${\sigma }_{m} ={\sigma }_{m}^{\mathrm{^{\prime}}}/\left(\frac{1}{nm}\sum_{k=1}^{nm}{||A\left({m}_{k}\right)||}^{2}\right)$$

where ${S}_{G}^{m}$ is defined as the microbe GIP kernel similarity matrix; ${\sigma }_{m}^{\mathrm{^{\prime}}}$ is a trade-off parameter and we set ${\sigma }_{m}^{\mathrm{^{\prime}}}=1$ in the experiments; parameter ${\sigma }_{m}$ is applied to tune-up bandwidth of GIP kernel, which can be updated by the Eq. (3).

Similarly, we also obtained the disease GIP kernel similarity as follows:

4 $${S}_{G}^{d}\left({d}_{p}, {d}_{q}\right)=exp\left(-{\sigma }_{d}{||A\left({d}_{p}\right)-A\left({d}_{q}\right)||}^{2}\right)$$

5 $${\sigma }_{d} ={\sigma }_{d}^{\mathrm{^{\prime}}}/\left(\frac{1}{nd}\sum_{k=1}^{nd}{||A\left({d}_{k}\right)||}^{2}\right)$$

where ${S}_{G}^{d}$ is defined as the disease GIP kernel similarity matrix; ${\sigma }_{d}^{\mathrm{^{\prime}}}$ is a trade-off parameter and we set ${\sigma }_{d}^{\mathrm{^{\prime}}}=1$ in the experiments; parameter ${\sigma }_{d}$ is applied to tune-up bandwidth of GIP kernel, which can be updated by the Eq. (5).

#### HIP similarity for microbes and diseases

In this work, inspired by the Jiang et al.' s work [38], we introduced HIP similarity to measure the interaction profile similarity between microbe pairs from the training adjacency matrix $A$. For HIP similarity, two microbes will have a lower similarity if they have more different corresponding values in the interaction profiles. Further, the HIP similarity of microbes ${m}_{i}$ and ${m}_{j}$ is defined as follows:

6 $${S}_{H}^{m}\left({m}_{i},{m}_{j}\right)=1-\frac{\left|A\left({m}_{i}\right)!=A\left({m}_{j}\right)\right|}{\left|A\left({m}_{i}\right)\right|}$$

where ${S}_{H}^{m}$ denotes the microbe HIP similarity matrix; $\left|\cdot \right|$ denotes the number of elements in the interaction profile.

Similarly, based on the interaction profiles of diseases, the HIP similarity of diseases can be calculated as follows:

7 $${ S}_{H}^{d}\left({d}_{p},{d}_{q}\right)=1-\frac{\left|A\left({d}_{p}\right)!=A\left({d}_{q}\right)\right|}{\left|A\left({d}_{p}\right)\right|}$$

where ${S}_{H}^{d}$ denotes the disease HIP similarity matrix.

### MDAKRLS for microbe-disease association prediction

Regularized least squares (RLS) and its extended versions are popular machine learning methods. In this work, to boost the predictable performance, a novel predict model called MDAKRLS is proposed to calculate the relevance scores between microbes and diseases by integrating the Kronecker product and RLS method.

We first obtained the Kronecker Gaussian similarity between microbe-disease pairs by the GIP similarity matrix of microbes and diseases. Specifically, we use the following equation to define the similarity between the microbe-disease pairs $\left(m\left(i\right),d\left(p\right)\right)$ and $\left(m\left(j\right),d\left(q\right)\right)$:

8 $${K}_{G}\left(\left(m\left(i\right),d\left(p\right)\right),\left(m\left(j\right),d\left(q\right)\right)\right)={S}_{G}^{m}\left(m\left(i\right),m\left(j\right)\right)*{S}_{G}^{d}\left(d\left(p\right),d\left(q\right)\right)$$

where ${S}_{G}^{m}$ and ${S}_{G}^{d}$ represent the GIP similarity matrix of microbes and diseases defined above, respectively. Let $N=nd\times nm$ which represents the number of microbe-disease pairs. The above equation can be represented by the Kronecker product as follows:

9 $${K}_{G}={S}_{G}^{m}\otimes {S}_{G}^{d}$$

where ${K}_{G }\epsilon {R}^{N\times N}$ is defined as the Kronecker Gaussian similarity of microbe-disease pairs. In the same manner, the Kronecker Hamming similarity matrix ${K}_{H }\epsilon {R}^{N\times N}$ of microbe-disease pairs can be measured:

10 $${K}_{H}={S}_{H}^{m}\otimes {S}_{H}^{d}$$

For a better description, in this work, we set $X=\left({x}_{1},{x}_{2},\dots ,{x}_{i},\dots ,{x}_{N}\right)$, where ${x}_{i}$ denotes the $i$ th microbe-disease pair. $vec\left(Y\right)=\left({y}_{1},{y}_{2},\dots ,{y}_{i},\dots ,{y}_{N}\right)$, where $Y {\epsilon R}^{nd\times nm}$ denotes the training microbe-disease adjacency matrix in the process of forecasting; $vec\left(\cdot \right)$ is a vector operator that stacks the elements of all columns into a vector; ${y}_{i}\epsilon \{0, 1\}$ denotes the corresponding label of microbe-disease pair ${x}_{i}$. The biological problem of predicting disease-related microbes can be transformed to learn a mapping function ${f}_{G}$ and calculate a corresponding association score. $vec\left({F}_{G}\right)=\left({f}_{G}\left({x}_{1}\right),{f}_{G}\left({x}_{2}\right),\dots ,{f}_{G}\left({x}_{i}\right),\dots ,{f}_{G}\left({x}_{N}\right)\right)$, where ${F}_{G}$ denotes the prediction score matrix based on the Kronecker Gaussian similarity; ${f}_{G}\left({x}_{i}\right)$ represents the prediction score of microbe-disease pair ${x}_{i}$ obtained by prediction function ${f}_{G}$.

In further work, first, we constructed the Kronecker regularized least squares [39] based on the Kronecker Gaussian similarity to solve the microbe-disease prediction problem. The objective function based on the Tikhonov minimization problem is formulated as follows:

11 $$J\left({f}_{G}\right)=\frac{1}{2}\sum_{i=1}^{N}{\left({y}_{i}-{f}_{G}\left({x}_{i}\right)\right)}^{2}+\frac{{\sigma }_{G}}{2}{\parallel {f}_{G}\parallel }_{k}^{2}$$

where ${\sigma }_{G}>0$ is a regularization coefficient used to adjust the regularization term and loss function of the objective function; ${\parallel {f}_{G}\parallel }_{k}$ is the norm of mapping function ${f}_{G}$ in Reproducing Kernel Hilbert Space (RKHS) [40] associated to the kernel $k$. Based on the classical Representer Theorem [41], the solution of the Tikhonov regularization problem exists in the RKHS and can be calculated as follows:

12 $${f}_{G}\left({x}_{i}\right)=\sum_{j=1}^{N}{\alpha }_{j}{K}_{G}\left({x}_{i},{x}_{j}\right)$$

According to the previous studies [37, 42], the optimal solution of the objective function can be further calculated as follows:

13 $$vec\left({F}_{G}\right)={K}_{G}{({K}_{G}+{\sigma }_{G} I)}^{-1}vec\left(Y\right)$$

where $I$ denotes the identity matrix.

Eigen decompositions were implemented on the GIP similarity matrix ${S}_{G}^{m}$ of microbes and GIP similarity matrix ${S}_{G}^{d}$ of diseases. We can get ${S}_{G}^{m}$=${V}_{G}^{m}{\Lambda }_{G}^{m}{{V}_{G}^{m}}^{\rm T}$ and ${S}_{G}^{d}$=${V}_{G}^{d}{\Lambda }_{G}^{d}{{V}_{G}^{d}}^{\rm T}$, respectively. According to the property of the Kronecker product, we can obtain the ${K}_{G}={S}_{G}^{m}\otimes {S}_{G}^{d}={V}_{G}{\Lambda }_{G}{{V}_{G}}^{\rm T}$, where ${V}_{G}={V}_{G}^{m}\otimes {V}_{G}^{d}$ and ${\Lambda }_{G}={\Lambda }_{G}^{m}\otimes {\Lambda }_{G}^{d}$. Then, we can transform the Eq. (13) as follows:

14 $$\begin{aligned} vec\left({F}_{G}\right)=&{V}_{G}{\Lambda }_{G}{{V}_{G}}^{\rm T}{\left({V}_{G}{\Lambda }_{G}{{V}_{G}}^{\rm T}+{\sigma }_{G} I\right)}^{-1}vec\left(Y\right)\\ =&{V}_{G}{\Lambda }_{G}{\left({\Lambda }_{G}+{\sigma }_{G} I\right)}^{-1}{{V}_{G}}^{\rm T}vec\left(Y\right)\end{aligned}$$

According to another property of the Kronecker product [43], $\left({N}^{\rm T} {\otimes} M\right)vec\left(C\right)=vec\left(MCN\right)$, Eq. (14) can be rewritten as follows:

$$vec\left({F}_{G}\right)={(V}_{G}^{m}\otimes {V}_{G}^{d})\left({\Lambda }_{G}^{m}\otimes {\Lambda }_{G}^{d}\right){\left({\Lambda }_{G}^{m}\otimes {\Lambda }_{G}^{d}+{\sigma }_{G} I\right)}^{-1}({{V}_{G}^{m}}^{\rm T}\otimes {{V}_{G}^{d}}^{\rm T})vec\left(Y\right)$$

$$={(V}_{G}^{m}\otimes {V}_{G}^{d})\left({\Lambda }_{G}^{m}\otimes {\Lambda }_{G}^{d}\right){\left({\Lambda }_{G}^{m}\otimes {\Lambda }_{G}^{d}+{\sigma }_{G} I\right)}^{-1}vec({{V}_{G}^{d}}^{\rm T}Y{V}_{G}^{m})$$

$$={(V}_{G}^{m}\otimes {V}_{G}^{d})vec({X}_{G})$$

15 $$=vec\left({V}_{G}^{d}{X}_{G}{{V}_{G}^{m}}^{\rm T}\right)$$

Finally, we will obtain the score matrix based on the Kronecker Gaussian similarity by the following equation:

16 $${F}_{G}^{*}={V}_{G}^{d}{X}_{G}{{V}_{G}^{m}}^{\rm T}$$

where $$vec\left({X}_{G}\right)=\left({\Lambda }_{G}^{m}\otimes {\Lambda }_{G}^{d}\right){\left({\Lambda }_{G}^{m}\otimes {\Lambda }_{G}^{d}+{\sigma }_{G} I\right)}^{-1}vec\left({{V}_{G}^{d}}^{\rm T}Y{V}_{G}^{m}\right).$$

In addition, we also can get another objective function and optimal solution based on the Kronecker Hamming similarity in a similar manner:

17 $$J\left({f}_{H}\right)=\frac{1}{2}\sum_{i=1}^{N}{\left({y}_{i}-{f}_{H}\left({x}_{i}\right)\right)}^{2}+\frac{{\sigma }_{H}}{2}{\parallel {f}_{H}\parallel }_{k}^{2}$$

18 $$vec\left({F}_{H}\right)={K}_{H}{({K}_{H}+{\sigma }_{H} I)}^{-1}vec\left(Y\right)$$

We implemented eigen decompositions on the HIP similarity matrix ${S}_{H}^{m}$ of microbes and HIP similarity matrix ${S}_{H}^{d}$ of diseases, and obtained the second score matrix based on the Kronecker Hamming similarity:

19 $${F}_{H}^{*}={V}_{H}^{d}{X}_{H}{{V}_{H}^{m}}^{\rm T}$$

where $$vec\left({X}_{H}\right)=\left({\Lambda }_{H}^{m}\otimes {\Lambda }_{H}^{d}\right){\left({\Lambda }_{H}^{m}\otimes {\Lambda }_{H}^{d}+{\sigma }_{H} I\right)}^{-1}vec\left({{V}_{H}^{d}}^{\rm T}Y{V}_{H}^{m}\right).$$

After obtaining the prediction matrix ${F}_{G}^{*}$ and ${F}_{H}^{*}$ based on the two different Kronecker similarities, respectively, we obtain the final prediction matrix by integrating their contributions as follows:

20 $${F}^{*}=w\cdot {F}_{G}^{*}+\left(1-w\right)\cdot {F}_{H}^{*}$$

where $w$ is a trade-off parameter. Eventually, we will obtain the score matrix ${F}^{*}$. In the future research, the association with the high score will have a priority to be verified by biological experiment.

## Results and discussion

### Evaluation metrics

To measure the reliability and effectiveness of the proposed method, in the same experimental conditions, we implemented our method and reran the other five state-of-the-art computational methods for comparison, under 5-CV and global LOOCV framework. Notably, the GIP kernel similarity and HIP similarity of microbes and diseases should be recalculated in every round of the global LOOCV and 5-CV framework.

Specifically, in the global LOOCV framework, all of the microbe-disease pairs without associations were used as candidate samples, each of the known MDA was treated as a testing sample and the rest of the known MDAs were treated as a training set to conduct experiments. We can obtain the rank of every testing sample by comparing it with candidate samples. To visualize the prediction performance, 1-specificity (false positive rates) and sensitivity (true positive rates) were calculated to plot the receiver operating characteristic (ROC) curves by setting different thresholds. For convenient observation, we calculated the area under the ROC curve (AUC) values to measure the ability of prediction method.

In the validation framework of 5-CV, all observed microbe-disease associations are randomly split into 5 subsets. Each of the 5 subsets is specified as an independent testing set and the rest of the 4 subsets are regarded as training sets. To weaken potential experimental bias caused by random sample division, the process of the experiment of every method was performed 100 times. Furthermore, the corresponding 1-specificity and sensitivity were obtained for plotting the ROC curves. The corresponding AUC values were also calculated for evaluation. The AUC value of 1 means best prediction, while the AUC value of 0.5 indicates random prediction.

### Parameter sensitivity analysis

There are three parameters (${\sigma }_{G}$, ${\sigma }_{H}$ and $w$) in our model. In general, the prediction performance of the model depends on some parameters, and different scale values of the parameter will produce different prediction results. Here, to explore the properties of the proposed method and the influences of parameter and find the optimal parameter, we calculated the AUCs and made some comparison experiments with different initial parameters under the 5-CV and LOOCV frameworks.

**Fig. 2:**
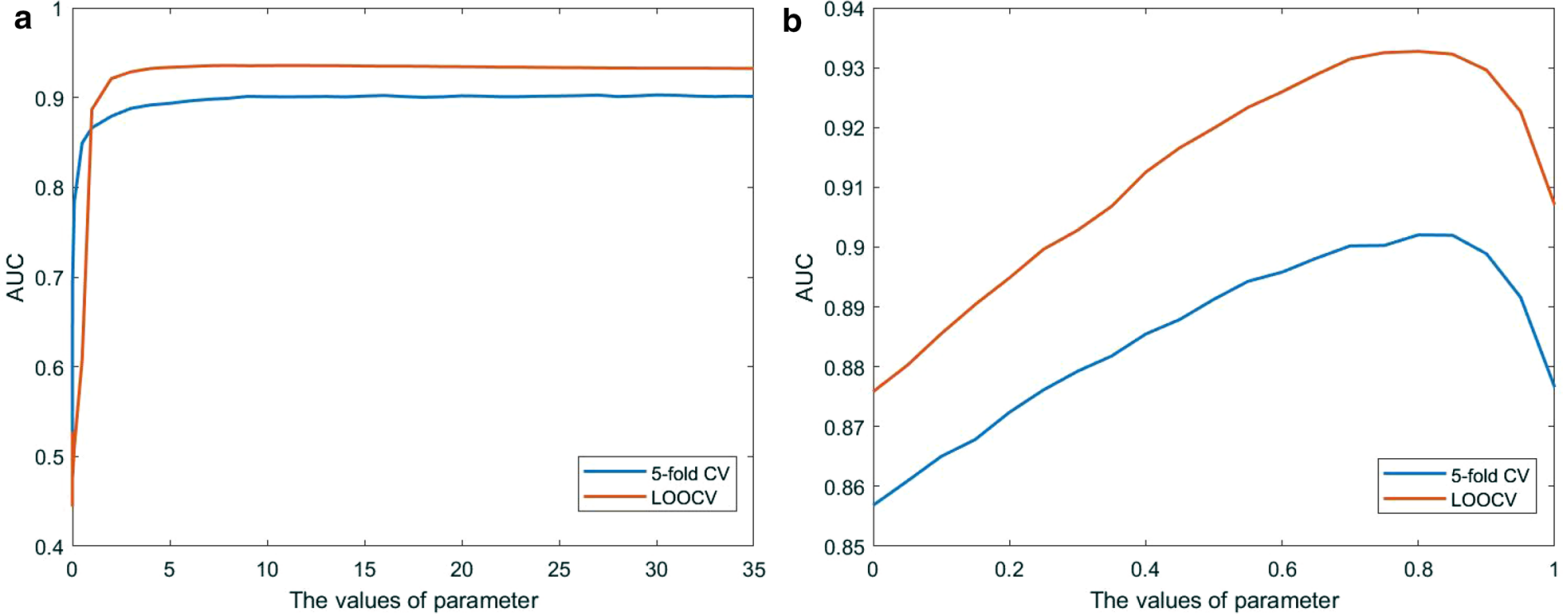
**a** The relationship between parameters ${\sigma }_{G}$ and ${\sigma }_{H}$ and AUC value under the 5-CV and LOOCV frameworks. **b** The relationship between parameter $w$ and AUC value under the 5-CV and LOOCV frameworks

${\sigma }_{G}$ and ${\sigma }_{H}$ are self-tuned parameters of MDAKRLS. To promote robust performance and simplify the complex problem, we set the same variable value for parameters ${\sigma }_{G}$ and ${\sigma }_{H}$. The experimental results of the parameters have been shown in Fig. 2a. From the figure, the average AUC of MDAKRLS is greatly enhanced when the parameter increases from 0 to 5, and the performance remains almost unchanged as the value of the parameter increases from 5 to 35 under two kinds of frameworks. Finally, the values of parameters ${\sigma }_{G}$ and ${\sigma }_{H}$ were set as 30 to obtain a stable and optimal prediction result. Then, we fixed ${\sigma }_{G}$ and ${\sigma }_{H}$, and adjusted the trade-off parameter $w$. The relationship between the AUC value and the parameter $w$ is shown in Fig. 2b. It can be seen that MDAKRLS will obtain the highest AUC whn $w=0.8$, indicating that the Kronecker Gaussian similarity can provide more effective information for prediction. Finally, we obtained the best parameters for the following analysis, which can achieve better performance. The average AUC value of 5-CV achieved by our proposed method based on the optimal parameters was 0.9023 ± 0.0015, and the AUC value of global LOOCV was 0.9327. The standard deviation and evaluation results demonstrate that used parameter values are reliable and robust for the proposed model.

### Comparison with other methods

**Fig. 3:**
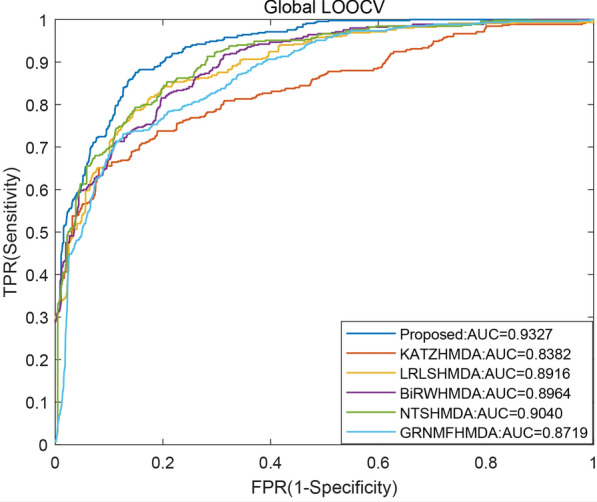
The AUC values and ROC curves of different methods under global LOOCV

**Fig. 4:**
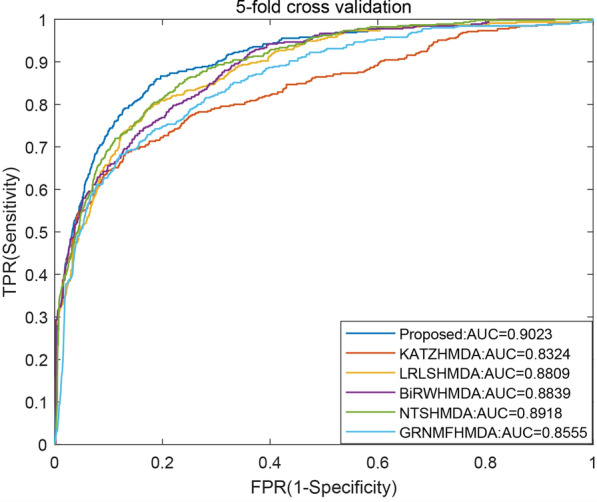
The average AUC values and ROC curves of different methods under 5-CV

To validate the effectiveness of MDAKRLS, we compared it with five state-of-the-art computational methods under the same experimental conditions, including KATZ measure (KATZHMDA) [19], Laplacian Regularized Least Squares (LRLSHMDA) [27], Bi-Random Walk (BiRWHMDA) [20], Network Topological Similarity (NTSHMDA) [21] and Graph Regularized Non-negative Matrix Factorization (GRNMFHMDA) [28] for human microbe–disease association prediction. Previous studies showed that these methods achieved effective prediction results. Here, we implemented the above 5 prediction methods for comparison under the global LOOCV and 5-CV frameworks on the same benchmark data set. The comparison results are shown in Figs. 3 and 4, respectively.

Specifically, Fig. 3 shows AUC values and ROC curves of different methods under the global LOOCV framework. It can be observed from the figure that the AUC values of five comparative methods are the following: KATZHMDA (0.8382), LRLSHMDA (0.8916), BiRWHMDA (0.8964), NTSHMDA (0.9040) and GRNMFHMDA (0.8719). Our method obtained the highest AUC value (0.9327), which is superior to the other five methods. Similarly, we compared all methods in the framework of 5-CV. The corresponding average AUC values and ROC curves of different methods have been shown in Fig. 4. As a result, the average AUC value of the proposed method is 0.9023, which performs better than KATZHMDA (0.8324), LRLSHMDA (0.8809), BiRWHMDA (0.8839), NTSHMDA (0.8918) and GRNMFHMDA (0.8555). The experimental results demonstrate that the proposed method is an effective and reliable prediction tool in inferring possible associations.

Practically, the traditional experiment-based methods are time-consuming, some computational methods were proposed to save time. It is necessary to improve calculation speed and prediction accuracy for developing new reliable computational methods. Thus, calculation speed is an important metric for performance evaluation of different computational methods. Therefore, it is a fresh perspective and we implemented a runtime analysis. Specifically, we compared the calculation speed between the proposed method and five state-of-the-art prediction methods under the global LOOCV and 5-CV frameworks. The comparisons of calculation speed analysis are shown in Table 1. Our proposed method obtained a faster average running time under the 5-CV framework. Moreover, the proposed method can use the shortest time for prediction under the global LOOCV framework. In brief, these results indicate the proposed method is reliable, effective and time-saving. It may be a useful tool for seeking disease-related new microbes.

**Table 1:** Comparison of calculation speed between proposed method and five state-of-the-art prediction methods

Method	5-fold CV	Global LOOCV
	Average running time (s)	Running time (s)
Proposed method	1.5604 $\pm$ 0.1290	7.4807
KATZHMDA	1.7240 $\pm$ 0.1190	8.6369
LRLSHMDA	1.8275 $\pm$ 0.2236	29.1313
BiRWHMDA	1.6293 $\pm$ 0.1057	11.7000
GRNMFHMDA	1.9593 $\pm$ 0.1669	30.1469
NTSHMDA	1.8302 + -0.8365	19.4239

### Case studies

To further access the practical effect of the proposed method in inferring associated microbes with a disease without any known associated microbes, case study analysis [28, 44] was implemented on the MDAKRLS. For a given disease, we removed all known microbe associations in the HMDAD. Then, the proposed method was trained on the rest of the known associations and tested on the candidate microbe samples to seek the disease-related microbes, it can guarantee the independence between training data sets and validation data sets. In other words, the prediction model only depends on the rest of the known association information and the similarity measures of the training data sets. Specifically, in the microbe-disease adjacency matrix $A$, we converted all 1 to 0 for a given disease and ranked all microbe samples based on the prediction scores. The top ranked microbes will be further verified by the relevant literature and the method will be effective if the top prediction results have more verified microbes. To reveal the pathological relationship of diseases and microbes, in the framework of the MDAKRLS, we implemented independent case studies on two kinds of important human diseases: asthma and inflammatory bowel disease (IBD). It should be noted that we assume that the genus of this microbe will be associated with the disease if the microbe is associated with the disease when we validate microbes [21, 45].

Asthma is a common chronic inflammatory disease, which has substantial morbidity. According to statistics, more than 300 million patients were affected by asthma worldwide [8]. In this study, the top 20 prediction results of asthma-related microbes are tabulated in Table 2. In the prediction list, there are some predictions have been validated by the HMDAD, the rest could be validated by the previously published biological and medical literature for asthma-related microbes. Finally, 19 of the top 20 prediction microbes could be manually verified that they are related to asthma patients. For example, *Actinobacteria*, *Firmicutes* and *Bacteroides* have lower proportions in all sputum samples of asthmatic patients, while *Proteobacteria* and *Staphylococcus aureus* were higher [46, 47]. Moreover, *Clostridium difficile* colonized at 1 month of age, which was closely related to asthma at 6 to 7 years of age [48]. The clustering results of bacterial composition showed *Enterobacteriaceae* family were more abundant in healthy people, while *Lachnospiraceae* and *Bifidobacterium* were more abundant in the asthma patients [49]. In addition, in a study about children and infants, the fecal colonization of *Clostridium coccoides* subcluster XIVa species and *Bacteroides fragilis* subgroup can be served as early indicators, which will be good for the prevention of asthma [50]. *Lactobacillus* has been shown to be beneficial for children with asthma [51].

**Table 2:** Prediction results of the top 20 associated microbes with asthma

Rank	Microbe	Evidence	Score
1	Proteobacteria	Confirmed by HMDAD	0.0840
2	Firmicutes	PMID:23265859	0.0698
3	Clostridium difficile	PMID:21872915	0.0687
4	Bacteroidetes	Confirmed by HMDAD	0.0683
5	Prevotella	Confirmed by HMDAD	0.0623
6	Helicobacter pylori	Confirmed by HMDAD	0.0571
7	Clostridium coccoides	PMID:21477358	0.0506
8	Actinobacteria	PMID:23265859	0.0503
9	Staphylococcus aureus	PMID:18822123	0.0450
10	Lachnospiraceae	PMID:28912020	0.0411
11	Lactobacillus	PMID:20592920	0.0388
12	Clostridia	Unconfirmed	0.0367
13	Enterobacteriaceae	PMID:28947029	0.0349
14	Bacteroides	PMID:18822123	0.0336
15	Veillonella	PMID:25329665	0.0301
16	Haemophilus	Confirmed by HMDAD	0.0297
17	Fusobacterium	PMID:27838347	0.0285
18	Stenotrophomonas maltophilia	PMID:16351036	0.0269
19	Bifidobacterium	PMID:24735374	0.0260
20	Bacteroides vulgatus	PMID:28966614	0.0250

IBD is a chronic disabling gastrointestinal disease with a continually increasing incidence, which is a worldwide health-care problem [9]. Similar to asthma, the top 20 prediction results of inflammatory bowel disease (IBD)-related microbes are tabulated in Table 3. In the prediction list, based on the HMDAD and recently published biological and medical literature for IBD-related microbes, 19 of the top 20 prediction microbes could be manually verified that they are related to the IBD patients. For example, previous studies showed *Bacteroidetes*, *Prevotella* and *Firmicutes* were associated with the formation of IBD [52, 53]. *Clostridium difficile* can aggravate flares of IBD, resulting in mortality and morbidity [54]. There is a negative relevant relation between IBD and *Helicobacter pylori* [55]. Compared with healthy people, *Clostridium coccoides* was less represented in active IBD patients [56]. In the salivary microbiota of IBD patients, *Haemophilus*, *Veillonella* and *Prevotella* were found that can largely contribute to dysbiosis [57]. In addition, in the faeces of IBD patients, the proportion of *Lactobacillus* increased, while *Bifidobacterium* decreased [58].

**Table 3:** Prediction results of the top 20 related microbes with IBD

Rank	Microbe	Evidence	Score
1	Proteobacteria	Confirmed by HMDAD	0.0820
2	Bacteroidetes	PMID:25307765	0.0798
3	Prevotella	PMID:25307765	0.0732
4	Firmicutes	PMID:25307765	0.0703
5	Clostridium difficile	PMID:24838421	0.0692
6	Helicobacter pylori	PMID:22221289	0.0684
7	Clostridium coccoides	PMID:19235886	0.0508
8	Staphylococcus aureus	PNID:19809406	0.0454
9	Haemophilus	PMID:24013298	0.0401
10	Lactobacillus	PMID:26340825	0.0389
11	Clostridia	PMID:25307765	0.0370
12	Actinobacteria	Confirmed by HMDAD	0.0370
13	Enterobacteriaceae	PMID:24629344	0.0351
14	Bacteroides	PMID:25307765	0.0336
15	Staphylococcus	PMID:28174737	0.0306
16	Veillonella	PMID:28842640	0.0301
17	Lachnospiraceae	Confirmed by HMDAD	0.0291
18	Fusobacterium	PMID:25307765	0.0282
19	Stenotrophomonas maltophilia	Unconfirmed	0.0271
20	Bifidobacterium	PMID:24478468	0.0260

In addition, we also implemented case studies for three metabolic diseases including Obesity, Type 1 diabetes and Type 2 diabetes (see Additional file 1). Case studies indicate if one of the 39 human diseases does not have any known related microbes in the HMDAD, MDAKRLS can calculate the possibility of association between the disease and 292 microbes. The proposed method may be an effective tool for seeking disease-related possible new microbes. Then we further used MDAKRLS to rank all candidate microbes for all the diseases involved in HMDAD (see Additional file 2). We hope that the prediction list can provide aid, and more and more potential microbe-disease pairs could be verified by clinical or biological experiment observation.

## Conclusion

Identifying of MDAs could help us better understand the pathogenesis of human diseases, which is also useful for the prevention, diagnosis and treatment of human diseases. In this study, we developed a novel computational method called MDAKRLS based on the Kronecker regularized least squares. Firstly, we not only calculated the Kronecker Gaussian similarity of microbe-disease pairs through the GIP kernel similarity of microbes and diseases, but also obtained the Kronecker Hamming similarity by the HIP similarity. Then, we developed the Kronecker regularized least squares based on the Kronecker product and RLS method to calculate the correlation scores of MDAs. A comparison of calculation speed showed our method has the advantage of fast calculating speed. The evaluation results of the 5-CV and the global LOOCV framework demonstrated that MDAKRLS improved calculation accuracy and had a reliable prediction performance. In addition, case studies of IBD and asthma further indicated that MDAKRLS can effectively discover potential associations.

Several critical factors that make MDAKRLS has a reliable prediction performance. Firstly, different from some methods only using the GIP kernel similarity for prediction, we also introduced the HIP similarity to measure the similarities of microbes and diseases. Secondly, we used the Kronecker product to construct two kinds of Kronecker similarities of microbe-disease pairs, which is complementary and can effectively mine the topological structure information of the network. Thirdly, In the process of solving the Tikhonov minimization problem, we introduced eigen decompositions to reduce the computational complexity. Kronecker regularized least squares is a machine learning-based method and uses fewer model parameters, thus saving time and improving robust performance. Of course, MDAKRLS needs to be improved in future work, such as some prior information of microbes or diseases could be introduced to improve the prediction performance; the insufficient number of experimentally verified MDAs limits the performance and development of the computational model.

The development of a reasonable and effective calculation model is conducive to the study of the microbial community. MDAKRLS has a good transplantation character, which is easily implemented to solve similar biological problems. The insufficient number of experimentally verified MDAs limits the performance and development of the computational model. At present, most disease-related microbes remain unknown in HMDAD. Therefore, it will be feasible and be of great practical significance to develop prediction algorithms that can effectively overcome the data sparsity problem. In addition, it is necessary to add more experimentally verified MDAs to improve the database, which can provide a foundation for improving the performance of computational method. We hope that our method could help biomedical researchers to carry out follow-up studies, and more and more potential microbe-disease associations could be verified by clinical or biological experimental observation.

## Supplementary Information

**Additional file 1.** The prediction results of the top 20 associated microbes with Obesity, Type 1 diabetes and Type 2 diabetes.

**Additional file 2.** We further used MDAKRLS to rank all candidate microbes for all the diseases involved in HMDAD. The prediction results may help biomedical researchers conduct experimental validation and follow-up research.

## Data Availability

The data set analyzed during the current study can be available at: http://www.cuilab.cn/hmdad.

## Abbreviations

MDAs: Microbe-disease associations
IBD: Inflammatory bowel disease
HMDAD: Human microbe–disease association database
GIP: Gaussian interaction profile
HIP: Hamming interaction profile
5-CV: 5-fold cross-validation
LOOCV: Leave-one-out cross-validation
ROC: Receiver operating characteristic
AUC: Area under the ROC curve
